# Supplementary material for: Genetically determined serum urate levels and cardiovascular and other diseases in UK Biobank cohort: A phenome-wide mendelian randomization study
Source: PLoS Med. 2019 Oct 18;16(10):e1002937. doi: 10.1371/journal.pmed.1002937 (PMC6799886; doi:10.1371/journal.pmed.1002937)
Supplement: S1 Text — (DOCX) [file pmed.1002937.s002.docx]

**S1 Text**

**UK Biobank data**

***Genotype data*** - Genotyping, quality control and genotype imputation were conducted by the UK Biobank team prior to the data release and the exact procedure is described by Bycroft *et al* [1]*.* The initial 50 000 participants were genotyped by the Affymetrix UK BiLEVE Axiom array and the remaining 45 000 participants were genotyped by the Affymetrix UK Biobank Axiom array. Genotype imputation was performed based on a merged reference panel of the Haplotype Reference Consortium (HRC) [2] and the UK10K haplotype resources [3], and the classical allelic variations at the MHC region were further imputed by using an additional multi-population reference panel [4]. For quality control, a list of field variables was made available by the UK Biobank to indicate the genotype quality, population structure, and genetic relatedness.

***Phenotype data*** - A variety of national health systems and sources were used by the UK Biobank to follow up the disease diagnosis, cancer occurrence, and causes of death among the enrolled participants. Currently, there are three main different types of health records (i.e. hospital inpatient episodes, cancer registry data and death registry data) that have been incorporated into the central database. The coding for clinical diagnoses in these datasets followed the World Health Organization’s International Classification of Diseases (ICD) coding systems but used different ICD versions (ICD-10 or ICD-9) according to the date of the record. Primary and/or secondary ICD codes are available in the hospital inpatient data and/or death registry data to classify the main causes and contributory causes of the event of hospitalization and/or death respectively.

**Study population and quality control**

In order to minimize the influence of the diverse population structure in UK Biobank, our study was constrained to a subset of unrelated White British subjects with high quality genotype data. The metrics used for genotype quality control (QC) were based on the data fields created by the UK Biobank. Samples that were identified as a sex mismatch, outliers with high heterozygosity or with high missing rate, putative aneuploidy in sex chromosome, individuals with excess relatives, or non-White British ancestry were all excluded from the analysis. The largest possible subset of individuals without relatedness were identified using an algorithm implemented in the R package “*i-graph (v1.0.1)*” developed by Bycroft and colleagues [1].

**Other data sources**

**GUGC:** Data on the genetic associations with serum urate levels were obtained from the Global Urate Genetic Consortium (GUGC) [5]. In brief, the GUGC performed meta-analysis of 48 genome-wide association studies (GWAS), totalling 110,347 individuals to assess common variants associated with serum urate levels in European origin. Genotyping was performed on genome-wide chips, and imputation was conducted using HapMap 2 data as the reference.

**GLGC:** Data on the genetic associations with plasma lipid levels were obtained from the Global Lipids Genetic Consortium (GLGC) [6].In brief, the GLGC performed a meta-analysis of 46 lipid GWAS and examined subjects of European ancestry, including 94,595 individuals from 23 studies genotyped with GWAS arrays and 93,982 individuals from 37 studies genotyped with the Metabochip array.

**GIANT:** Data on the genetic associations with body mass index (BMI) and waist to hip ratio (WHR) were obtained from the Genetic Investigation of ANthropometric Traits (GIANT) consortium [7]. In brief, GIANT performed a meta-analysis of 51 GWAS assessing common variants associated with BMI in over 170,000 individuals of European descent. Genotyping was performed using commercially available Affymetrix or Illumina genotyping arrays or custom Perlegen arrays.

**MAGIC:** Data on the genetic associations with plasma glucose was obtained from the Meta-Analyses of Glucose and Insulin-related traits (MAGIC) Consortium [8]. MAGIC was a genome wide association study (GWAS) that sought to identify genetic determinants of glycemic and metabolic traits. The association between genetic variants and the change in FG (mmol/L) was assessed in 133,010 and 42,854 non-diabetic European individuals. Genotyping was performed using the Metabochip.

**ICBP:** Data on the genetic association with SBP and DBP was obtained from the International Consortium for Blood Pressure (ICBP) [9]. The ICBP-GWAS evaluated associations between 2.5 million genotyped or imputed single nucleotide polymorphisms (SNPs) and SBP and DBP in European ancestry from 29 studies. All studies with GWAS data performed genotyping using commercially available arrays with >300,000 SNPs and were imputed to the HapMap reference panels.

**CARDIoGRAMplusC4D:** Data on the genetic association with the risk of CAD was obtained from the Coronary ARtery DIsease Genome wide Replication and Meta-analysis [CARDIoGRAM] plus The Coronary Artery Disease [C4D] Genetics (CARDIoGRAMplusC4D) Consortium. Briefly, the CARDIoGRAMplusC4D Consortium performed a meta-analysis of 63,746 cases and 130,681 controls. Genotyping was performed using the Metabochip, which is a custom iSELECT chop (Illumina).

**ISGC:** Data on the genetic associations with ischaemic stroke was obtained from the Ischemic stroke Genetic Consortium (ISGC) [10]. They meta-analysed 12 individual genome-wide association studies comprising 10,307 cases and 19,326 controls imputed to the 1000 Genomes (1 KG) phase I reference panel. Genotyping was performed using the Sequenom iPLEX Gold chemistry and genotypes were called using SpectroCHIP array.

**MR-MOE**

Given many MR methods have been developed and each method assumes or performs best for a different model of pleiotropy in order to avoid cherry picking of MR results, we applied a machine learning approach (MR-MoE) to protect MR causal estimates from different patterns of horizontal pleiotropy. Specifically, the MR-MoE considered the 10 MR methods (as described below and in S1 Table), predicted the performance of each MR method in the context of different models of pleiotropy, and selected the method most likely to be correct for a specific causal analysis. Full details of the MR-MoE are available at <http://mrcieu.github.io/TwoSampleMR/#mr-moe-using-a-mixture-of-experts-machine-learning-approach> [11].

***Mean-based methods***: Four MR approaches (IVW fixed effects, IVW random effects, Egger fixed effects, Egger random effects) provide four mean-based estimators. The inverse variance weighted (IVW) fixed effects meta-analysis approach assumes that variants exhibit no horizontal pleiotropy. IVW random effects meta-analysis relaxes the horizontal pleiotropy assumption, allowing it to be present but balanced - such that it only leads to increased heterogeneity around the regression line without affecting the slope (and therefore not introducing bias). Fixed effects Egger regression relaxes the horizontal pleiotropy assumption further by allowing a non-zero intercept which essentially allows overall horizontal pleiotropy to be directional, where its total effect influences the outcome in a specific direction [12]. Random effects Egger regression further allows heterogeneity around the slope having accounted for overall directional horizontal pleiotropy [13], as long as the horizontal pleiotropy effects are not correlated with the SNP-exposure effects (also known as the INSIDE assumption) [12].

***Median-based methods***: This analytical approach takes the median effect of all available instruments [14]. The sample median method requires that half the instruments need to be valid to obtain unbiased estimate. The weighted median method allows stronger instruments to contribute more towards the estimate and obtain an estimates by weighting the contribution of each instrument by the inverse of its variance. The penalised weighted median estimator introduces a further weight to the instruments, penalising any instrument that contributes substantially towards the heterogeneity statistic. Together, this provides three median-based estimators [14].

***Mode-based methods***: The mode-based estimator clusters the instruments into groups based on similarity of causal effects, and returns the final causal effect estimate based on the cluster that has the largest number of instruments [15]. This provide three mode-based estimators: the simple mode is the unweighted mode of the empirical density function of causal estimates, the weighted mode is weighted by the inverse variance of the outcome effect, and the penalised weighted mode introduces a further weight to the instruments, penalising any instrument that contributes substantially towards the heterogeneity statistic.

**References**

1. Bycroft C, Freeman C, Petkova D, Band G, Elliott LT, Sharp K, et al. Genome-wide genetic data on~ 500,000 UK Biobank participants. bioRxiv. 2017.

2. McCarthy S, Das S, Kretzschmar W, Delaneau O, Wood AR, Teumer A, et al. A reference panel of 64,976 haplotypes for genotype imputation. Nat Genet. 2016;48(10):1279-83.

3. Walter K, Min JL, Huang J, Crooks L, Memari Y, McCarthy S, et al. The UK10K project identifies rare variants in health and disease. Nature. 2015;526(7571):82-90.

4. Dilthey A, Leslie S, Moutsianas L, Shen J, Cox C, Nelson MR, et al. Multi-population classical HLA type imputation. PLoS Comput Biol. 2013;9(2):e1002877.

5. Kottgen A, Albrecht E, Teumer A, Vitart V, Krumsiek J, Hundertmark C, et al. Genome-wide association analyses identify 18 new loci associated with serum urate concentrations. Nat Genet. 2013;45(2):145-54.

6. Willer CJ, Schmidt EM, Sengupta S, Peloso GM, Gustafsson S, Kanoni S, et al. Discovery and refinement of loci associated with lipid levels. Nat Genet. 2013;45(11):1274-83.

7. Shungin D, Winkler TW, Croteau-Chonka DC, Ferreira T, Locke AE, Magi R, et al. New genetic loci link adipose and insulin biology to body fat distribution. Nature. 2015;518(7538):187-96.

8. Manning AK, Hivert MF, Scott RA, Grimsby JL, Bouatia-Naji N, Chen H, et al. A genome-wide approach accounting for body mass index identifies genetic variants influencing fasting glycemic traits and insulin resistance. Nat Genet. 2012;44(6):659-69.

9. Ehret GB, Munroe PB, Rice KM, Bochud M, Johnson AD, Chasman DI, et al. Genetic variants in novel pathways influence blood pressure and cardiovascular disease risk. Nature. 2011;478(7367):103-9.

10. Malik R, Traylor M, Pulit SL, Bevan S, Hopewell JC, Holliday EG, et al. Low-frequency and common genetic variation in ischemic stroke: The METASTROKE collaboration. Neurology. 2016;86(13):1217-26.

11. Hemani G, Bowden J, Haycock P, Zheng J, Davis O, Flach P, et al. Automating Mendelian randomization through machine learning to construct a putative causal map of the human phenome. bioRxiv doi: https://doi.org/10.1101/173682. 2017.

12. Bowden J, Davey Smith G, Burgess S. Mendelian randomization with invalid instruments: effect estimation and bias detection through Egger regression. Int J Epidemiol. 2015;44(2):512-25.

13. Bowden J, Del Greco MF, Minelli C, Davey Smith G, Sheehan N, Thompson J. A framework for the investigation of pleiotropy in two-sample summary data Mendelian randomization. Stat Med. 2017;36(11):1783-802.

14. Bowden J, Davey Smith G, Haycock PC, Burgess S. Consistent Estimation in Mendelian Randomization with Some Invalid Instruments Using a Weighted Median Estimator. Genet Epidemiol. 2016;40(4):304-14.

15. Hartwig FP, Davey Smith G, Bowden J. Robust inference in summary data Mendelian randomization via the zero modal pleiotropy assumption. Int J Epidemiol. 2017;46(6):1985-98.
