# Supplementary material for: Genetically determined serum urate levels and cardiovascular and other diseases in UK Biobank cohort: A phenome-wide mendelian randomization study
Source: PLoS Med. 2019 Oct 18;16(10):e1002937. doi: 10.1371/journal.pmed.1002937 (PMC6799886; doi:10.1371/journal.pmed.1002937)
Supplement: S1 Table — MR, mendelian randomization. (DOCX) [file pmed.1002937.s004.docx]

**S1 Table. A summary of MR analytical approaches and their assumptions**

| **Method type** | **Methods** | **Assumptions** |
| --- | --- | --- |
| Mean-based methods | FE IVW^*^ | No horizontal pleiotropy |
|  | RE IVW^*^ | Balanced horizontal pleiotropy |
|  | FE Egger^*^ | No horizontal pleiotropy after accounting for directional pleiotropy |
|  | RE Egger^*^ | Balanced horizontal pleiotropy after accounting for directional pleiotropy |
|  | Simple mean | Only balanced and/or directional pleiotropy |
| Median-based methods | Simple median | At least half of the instruments are valid. |
|  | Penalised median | At least half the weight of the instruments is due to valid instruments, where each instrument that contributes to high heterogeneity is down weighted. |
|  | Weighted median | At least half the weight of the instruments is due to valid instruments. |
| Mode-based methods | Simple mode | After clustering instruments by causal estimates, the largest cluster is correct, weighting by the exposure variance. |
|  | Weighted mode | After clustering instruments by causal estimates, the largest cluster is correct, weighting by the exposure and outcome variances. |
|  | Penalised mode | After clustering instruments by causal estimates, the largest cluster is correct, where each instrument that contributes to high heterogeneity is down weighted. |

^*^Abbreviations: FE IVW: inverse variance weighted MR with fixed effects; RE IVW: inverse variance weighted MR with random effects; FE Egger: Egger MR with fixed effects; RE Egger: Egger MR with random effects.
