# Supplementary material for: Genetically determined serum urate levels and cardiovascular and other diseases in UK Biobank cohort: A phenome-wide mendelian randomization study
Source: PLoS Med. 2019 Oct 18;16(10):e1002937. doi: 10.1371/journal.pmed.1002937 (PMC6799886; doi:10.1371/journal.pmed.1002937)
Supplement: S2 Table — GWAS, genome-wide association study. (DOCX) [file pmed.1002937.s005.docx]

**S2 Table. A summary of genetic risk variants identified in previous urate GWAS.^*^**

| **SNP** | **Chr** | **Closest/GRAIL gene** | **Effect allele** | **Allele freq** | **beta** | **se** | **p-value** | **Pleiotropy** |
| --- | --- | --- | --- | --- | --- | --- | --- | --- |
| rs10821905 | 10 | *A1CF/ASAH2* | A | 0.824 | 0.053 | 0.007 | 3.45E-12 | No |
| rs1165151 | 6 | *SLC17A1/SLC17A3* | T | 0.549 | -0.092 | 0.005 | 4.52E-60 | No |
| rs12498742 | 4 | *SLC2A9/SLC2A9* | A | 0.232 | 0.380 | 0.006 | 0.00E+00 | No |
| rs1394125 | 15 | *UBE2Q2/NRG4* | A | 0.638 | 0.043 | 0.006 | 9.78E-11 | No |
| rs1471633 | 1 | *PDZK1/PDZK1* | A | 0.538 | 0.061 | 0.005 | 1.40E-26 | No |
| rs164009 | 17 | *QRICH2/PRPSAP1* | A | 0.387 | 0.029 | 0.006 | 7.06E-07 | No |
| rs17632159 | 5 | *TMEM171/TMEM171* | C | 0.697 | -0.038 | 0.006 | 2.00E-09 | No |
| rs17786744 | 8 | *STC1/STC1* | A | 0.410 | -0.031 | 0.005 | 8.82E-08 | No |
| rs2078267 | 11 | *SLC22A11/SLC22A11* | T | 0.452 | -0.078 | 0.006 | 8.73E-36 | No |
| rs675209 | 6 | *RREB1/RREB1* | T | 0.731 | 0.063 | 0.006 | 1.38E-21 | No |
| rs6770152 | 3 | *SFMBT1/MUSTN1* | T | 0.424 | -0.048 | 0.006 | 2.66E-16 | No |
| rs7188445 | 16 | *MAF/MAF* | A | 0.672 | -0.032 | 0.006 | 1.15E-07 | No |
| rs7224610 | 17 | *HLF/HLF* | A | 0.396 | -0.038 | 0.006 | 4.74E-11 | No |
| rs742132 | 6 | *LRRC16A/LRRC16A* | A | 0.294 | 0.035 | 0.006 | 1.90E-08 | No |
| rs10480300 | 7 | *PRKAG2/PRKAG2* | T | 0.727 | 0.032 | 0.006 | 9.37E-07 | Yes |
| rs11264341 | 1 | *TRIM46/PKLR* | T | 0.571 | -0.048 | 0.006 | 1.04E-14 | Yes |
| rs1171614 | 10 | *SLC16A9/SLC16A9* | T | 0.769 | -0.074 | 0.007 | 6.48E-23 | Yes |
| rs1178977 | 7 | *BAZ1B/MLXIPL* | A | 0.198 | 0.050 | 0.007 | 6.68E-12 | Yes |
| rs1260326 | 2 | *GCKR/GCKR* | T | 0.607 | 0.077 | 0.006 | 1.31E-40 | Yes |
| rs17050272 | 2 | *INHBB/INHBB* | A | 0.589 | 0.037 | 0.006 | 9.36E-09 | Yes |
| rs2079742 | 17 | *BCAS3/C17orf82* | T | 0.136 | 0.051 | 0.008 | 6.24E-09 | Yes |
| rs2231142 | 4 | *ABCG2/ABCG2* | T | 0.887 | 0.220 | 0.009 | 4.43E-116 | Yes |
| rs2307394 | 2 | *ORC4L/ACVR2A* | T | 0.303 | -0.035 | 0.006 | 7.26E-09 | Yes |
| rs2941484 | 8 | *HNF4G/HNF4G* | T | 0.553 | 0.049 | 0.006 | 3.91E-17 | Yes |
| rs3741414 | 12 | *INHBC/INHBE* | T | 0.755 | -0.071 | 0.007 | 9.79E-22 | Yes |
| rs478607 | 11 | *NRXN2/SLC22A12* | A | 0.153 | -0.048 | 0.007 | 5.31E-10 | Yes |
| rs642803 | 11 | *OVOL1/LTBP3* | T | 0.536 | -0.043 | 0.005 | 4.51E-14 | Yes |
| rs653178 | 12 | *ATXN2/PTPN11* | T | 0.483 | -0.036 | 0.005 | 2.45E-10 | Yes |
| rs6598541 | 15 | *IGF1R/IGF1R* | A | 0.645 | 0.044 | 0.006 | 5.20E-13 | Yes |
| rs7193778 | 16 | *NFAT5/NFAT5* | T | 0.150 | -0.047 | 0.008 | 2.36E-08 | Yes |
| rs729761 | 6 | *VEGFA/VEGFA* | T | 0.715 | -0.046 | 0.006 | 3.05E-12 | Yes |

*GWAS summary data were obtained from the Global Urate Genetic Consortium (GUGC); one SNP (rs164009) was included on the basis of its functional role in urate metabolism (encoding a protein involved in the regulation of purine synthesis).
