# Supplementary material for: Genetically determined serum urate levels and cardiovascular and other diseases in UK Biobank cohort: A phenome-wide mendelian randomization study
Source: PLoS Med. 2019 Oct 18;16(10):e1002937. doi: 10.1371/journal.pmed.1002937 (PMC6799886; doi:10.1371/journal.pmed.1002937)
Supplement: S3 Table — (DOCX) [file pmed.1002937.s006.docx]

**S3 Table. A summary of pleiotropic loci on urate and obesity traits.^*^**

| **SNP** | **Chr** | **Closest/GRAIL gene** | **Effect allele** | **BMI** | | | **WHR** | | | **Pleiotropy** |
| --- | --- | --- | --- | --- | --- | --- | --- | --- | --- | --- |
|  |  |  |  | **beta** | **se** | **p-value** | **beta** | **se** | **p-value** |  |
| rs2231142 | 4 | *ABCG2/ABCG2* | T | -0.081 | 0.017 | 2.16E-06 | 0.029 | 0.025 | 0.261 | Yes |
| rs7193778 | 16 | *NFAT5/NFAT5* | T | 0.339 | 0.072 | 2.17E-06 | 0.468 | 0.130 | 3.10E-04 | Yes |
| rs2941484 | 8 | *HNF4G/HNF4G* | T | 0.207 | 0.050 | 3.07E-05 | -0.204 | 0.088 | 0.020 | Yes |
| rs1260326 | 2 | *GCKR/GCKR* | T | -0.131 | 0.032 | 4.22E-05 | 0.130 | 0.045 | 0.004 | Yes |
| rs478607 | 11 | *NRXN2/SLC22A12* | A | -0.281 | 0.069 | 5.08E-05 | 0.152 | 0.119 | 0.200 | Yes |
| rs11264341 | 1 | *TRIM46/PKLR* | T | 0.192 | 0.050 | 1.40E-04 | -0.152 | 0.092 | 0.097 | Yes |
| rs642803 | 11 | *OVOL1/LTBP3* | T | 0.198 | 0.056 | 4.22E-04 | 0.133 | 0.077 | 0.084 | Yes |
| rs653178 | 12 | *ATXN2/PTPN11* | T | -0.233 | 0.067 | 4.93E-04 | 0.072 | 0.097 | 0.458 | Yes |
| rs6598541 | 15 | *IGF1R/IGF1R* | A | 0.194 | 0.057 | 6.89E-04 | 0.089 | 0.077 | 0.251 | Yes |
| rs1178977 | 7 | *BAZ1B/MLXIPL* | A | -0.179 | 0.060 | 0.003 | 0.360 | 0.086 | 2.84E-05 | Yes |
| rs1471633 | 1 | *PDZK1/PDZK1* | A | 0.091 | 0.039 | 0.021 | -0.069 | 0.072 | 0.340 | No |
| rs2079742 | 17 | *BCAS3/C17orf82* | T | -0.158 | 0.069 | 0.022 | -0.039 | 0.129 | 0.762 | No |
| rs3741414 | 12 | *INHBC/INHBE* | T | -0.060 | 0.039 | 0.128 | 0.008 | 0.056 | 0.881 | No |
| rs7224610 | 17 | *HLF/HLF* | A | -0.099 | 0.065 | 0.128 | -0.082 | 0.113 | 0.471 | No |
| rs17050272 | 2 | *INHBB/INHBB* | A | 0.099 | 0.066 | 0.133 | -0.016 | 0.124 | 0.896 | No |
| rs729761 | 6 | *VEGFA/VEGFA* | T | -0.087 | 0.058 | 0.136 | 0.261 | 0.104 | 0.012 | No |
| rs1171614 | 10 | *SLC16A9/SLC16A9* | T | 0.057 | 0.039 | 0.138 | 0.080 | 0.078 | 0.309 | No |
| rs2307394 | 2 | *ORC4L/ACVR2A* | T | 0.106 | 0.075 | 0.159 | -0.031 | 0.129 | 0.807 | No |
| rs1394125 | 15 | *UBE2Q2/NRG4* | A | -0.076 | 0.058 | 0.193 | -0.067 | 0.114 | 0.554 | No |
| rs12498742 | 4 | *SLC2A9/SLC2A9* | A | 0.010 | 0.007 | 0.203 | -0.001 | 0.013 | 0.952 | No |
| rs17786744 | 8 | *STC1/STC1* | A | 0.098 | 0.079 | 0.217 | 0.155 | 0.139 | 0.264 | No |
| rs10480300 | 7 | *PRKAG2/PRKAG2* | T | -0.103 | 0.084 | 0.222 | -0.172 | 0.150 | 0.252 | No |
| rs675209 | 6 | *RREB1/RREB1* | T | -0.046 | 0.043 | 0.280 | -0.206 | 0.079 | 0.009 | No |
| rs742132 | 6 | *LRRC16A/LRRC16A* | A | 0.075 | 0.075 | 0.320 | 0.174 | 0.131 | 0.185 | No |
| rs1165151 | 6 | *SLC17A1/SLC17A3* | T | -0.025 | 0.026 | 0.345 | 0.050 | 0.046 | 0.273 | No |
| rs6770152 | 3 | *SFMBT1/MUSTN1* | T | 0.039 | 0.051 | 0.448 | 0.044 | 0.090 | 0.625 | No |
| rs2078267 | 11 | *SLC22A11/SLC22A11* | T | -0.023 | 0.031 | 0.453 | -0.104 | 0.055 | 0.060 | No |
| rs10821905 | 10 | *A1CF/ASAH2* | A | -0.031 | 0.060 | 0.600 | 0.053 | 0.106 | 0.617 | No |
| rs17632159 | 5 | *TMEM171/TMEM171* | C | 0.027 | 0.069 | 0.694 | -0.016 | 0.126 | 0.901 | No |
| rs7188445 | 16 | *MAF/MAF* | A | 0.011 | 0.080 | 0.893 | -0.203 | 0.141 | 0.149 | No |
| rs164009 | 17 | *QRICH2/PRPSAP1* | A | -0.007 | 0.085 | 0.936 | -0.100 | 0.152 | 0.510 | No |

*GWAS summary data were obtained from the Genetic Investigation of ANthropometric Traits (GIANT) consortium.

Abbreviations: chr, chromosome; BMI, body mass index; WHR, waist to hip ratio.
