# Supplementary material for: Genetically determined serum urate levels and cardiovascular and other diseases in UK Biobank cohort: A phenome-wide mendelian randomization study
Source: PLoS Med. 2019 Oct 18;16(10):e1002937. doi: 10.1371/journal.pmed.1002937 (PMC6799886; doi:10.1371/journal.pmed.1002937)
Supplement: S4 Table — BP, blood pressure. (DOCX) [file pmed.1002937.s007.docx]

**S4 Table. A summary of pleiotropic loci on urate and blood pressure (BP).^*^**

| **SNP** | **Chr** | **Closest/GRAIL gene** | **Effect allele** | **DBP** | | | **SBP** | | | **Pleiotropy** |
| --- | --- | --- | --- | --- | --- | --- | --- | --- | --- | --- |
|  |  |  |  | **beta** | **se** | **p-value** | **beta** | **se** | **p-value** |  |
| rs653178 | 12 | *ATXN2/PTPN11* | T | 1.057 | 0.068 | 6.66E-54 | 0.585 | 0.068 | 1.16E-17 | Yes |
| rs642803 | 11 | *OVOL1/LTBP3* | T | 0.377 | 0.057 | 4.83E-11 | 0.269 | 0.057 | 2.85E-06 | Yes |
| rs2307394 | 2 | *ORC4L/ACVR2A* | T | 0.396 | 0.077 | 2.47E-07 | 0.091 | 0.077 | 0.235 | Yes |
| rs10480300 | 7 | *PRKAG2/PRKAG2* | T | 0.364 | 0.086 | 2.38E-05 | 0.453 | 0.086 | 1.45E-07 | Yes |
| rs729761 | 6 | *VEGFA/VEGFA* | T | 0.236 | 0.060 | 7.38E-05 | -0.040 | 0.060 | 0.506 | Yes |
| rs2941484 | 8 | *HNF4G/HNF4G* | T | 0.194 | 0.051 | 1.30E-04 | 0.155 | 0.051 | 0.002 | Yes |
| rs1178977 | 7 | *BAZ1B/MLXIPL* | A | 0.230 | 0.062 | 1.93E-04 | 0.026 | 0.062 | 0.676 | Yes |
| rs7193778 | 16 | *TRIM46/PKLR* | T | -0.081 | 0.073 | 0.268 | 0.347 | 0.073 | 2.19E-06 | Yes |
| rs11264341 | 1 | *BCAS3/C17orf82* | T | 0.146 | 0.052 | 0.005 | 0.173 | 0.052 | 8.20E-04 | Yes |
| rs2079742 | 17 | *NFAT5/NFAT5* | T | 0.136 | 0.070 | 0.054 | 0.257 | 0.070 | 2.52E-04 | Yes |
| rs6770152 | 3 | *SFMBT1/MUSTN1* | T | 0.154 | 0.052 | 0.003 | 0.119 | 0.052 | 0.022 | No |
| rs7188445 | 16 | *MAF/MAF* | A | -0.214 | 0.082 | 0.009 | -0.037 | 0.082 | 0.655 | No |
| rs7224610 | 17 | *HLF/HLF* | A | 0.174 | 0.067 | 0.009 | 0.202 | 0.067 | 0.002 | No |
| rs12498742 | 4 | *SLC2A9/SLC2A9* | A | 0.017 | 0.008 | 0.023 | 0.009 | 0.008 | 0.219 | No |
| rs1471633 | 1 | *PDZK1/PDZK1* | A | 0.080 | 0.040 | 0.048 | 0.041 | 0.040 | 0.313 | No |
| rs17786744 | 8 | *STC1/STC1* | A | 0.140 | 0.081 | 0.083 | -0.114 | 0.081 | 0.159 | No |
| rs17050272 | 2 | *INHBB/INHBB* | A | 0.111 | 0.068 | 0.099 | 0.063 | 0.068 | 0.354 | No |
| rs3741414 | 12 | *INHBC/INHBE* | T | 0.062 | 0.040 | 0.126 | 0.125 | 0.040 | 0.002 | No |
| rs1165151 | 6 | *SLC17A1/SLC17A3* | T | 0.041 | 0.027 | 0.129 | 0.069 | 0.027 | 0.010 | No |
| rs1171614 | 10 | *SLC16A9/SLC16A9* | T | 0.048 | 0.039 | 0.224 | 0.103 | 0.039 | 0.009 | No |
| rs17632159 | 5 | *TMEM171/TMEM171* | C | -0.063 | 0.071 | 0.368 | -0.066 | 0.070 | 0.349 | No |
| rs10821905 | 10 | *A1CF/ASAH2* | A | 0.046 | 0.061 | 0.447 | 0.174 | 0.061 | 0.004 | No |
| rs478607 | 11 | *NRXN2/SLC22A12* | A | 0.049 | 0.071 | 0.490 | 0.106 | 0.071 | 0.136 | No |
| rs2231142 | 4 | *ABCG2/ABCG2* | T | -0.012 | 0.018 | 0.497 | -0.050 | 0.018 | 0.005 | No |
| rs2078267 | 11 | *SLC22A11/SLC22A11* | T | 0.020 | 0.032 | 0.523 | 0.000 | 0.032 | 0.999 | No |
| rs164009 | 17 | *QRICH2/PRPSAP1* | A | 0.050 | 0.087 | 0.570 | -0.025 | 0.087 | 0.770 | No |
| rs1394125 | 15 | *UBE2Q2/NRG4* | A | 0.026 | 0.060 | 0.658 | 0.042 | 0.060 | 0.477 | No |
| rs6598541 | 15 | *IGF1R/IGF1R* | A | 0.023 | 0.059 | 0.698 | -0.029 | 0.059 | 0.618 | No |
| rs1260326 | 2 | *GCKR/GCKR* | T | -0.012 | 0.033 | 0.714 | 0.066 | 0.033 | 0.044 | No |
| rs675209 | 6 | *RREB1/RREB1* | T | 0.012 | 0.044 | 0.792 | -0.024 | 0.044 | 0.593 | No |
| rs742132 | 6 | *LRRC16A/LRRC16A* | A | 0.005 | 0.077 | 0.950 | 0.003 | 0.077 | 0.966 | No |

*GWAS summary data were obtained from the International Consortium for Blood Pressure (ICBP).

Abbreviations: chr, chromosome; SBP, systolic blood pressure; DBP, diastolic blood pressure.
