# Supplementary material for: Genetically determined serum urate levels and cardiovascular and other diseases in UK Biobank cohort: A phenome-wide mendelian randomization study
Source: PLoS Med. 2019 Oct 18;16(10):e1002937. doi: 10.1371/journal.pmed.1002937 (PMC6799886; doi:10.1371/journal.pmed.1002937)
Supplement: S5 Table — (DOCX) [file pmed.1002937.s008.docx]

**S5 Table. A summary of pleiotropic loci on urate and lipids.^*^**

| **SNPs** | **Chr** | **Closest/GRAIL gene** | **Effect allele** | **TC** | | | **LDL-c** | | | **HDL-c** | | | **Pleiotropy** |
| --- | --- | --- | --- | --- | --- | --- | --- | --- | --- | --- | --- | --- | --- |
|  |  |  |  | **beta** | **se** | **p-value** | **beta** | **se** | **p-value** | **beta** | **se** | **p-value** |  |
| rs1260326 | 2 | *GCKR/GCKR* | T | 0.665 | 0.047 | 6.67E-46 | 0.268 | 0.048 | 2.58E-08 | -0.147 | 0.045 | 1.24E-03 | Yes |
| rs653178 | 12 | *ATXN2/PTPN11* | T | -0.853 | 0.103 | 1.07E-16 | -0.631 | 0.106 | 2.32E-09 | -0.731 | 0.097 | 5.72E-14 | Yes |
| rs17050272 | 2 | *INHBB/INHBB* | A | -0.573 | 0.159 | 3.27E-04 | -0.668 | 0.162 | 3.84E-05 | 0.027 | 0.151 | 0.858 | Yes |
| rs642803 | 11 | *OVOL1/LTBP3* | T | -0.281 | 0.084 | 7.76E-04 | -0.270 | 0.086 | 0.002 | -0.342 | 0.079 | 1.54E-05 | Yes |
| rs3741414 | 12 | *INHBC/INHBE* | T | 0.118 | 0.059 | 0.046 | 0.224 | 0.061 | 2.18E-04 | -0.417 | 0.056 | 1.36E-13 | Yes |
| rs1178977 | 7 | *BAZ1B/MLXIPL* | A | 0.196 | 0.090 | 0.029 | -0.068 | 0.094 | 0.469 | -0.632 | 0.088 | 6.88E-13 | Yes |
| rs6770152 | 3 | *SFMBT1/MUSTN1* | T | -0.302 | 0.108 | 0.005 | -0.244 | 0.110 | 0.027 | -0.142 | 0.102 | 0.165 | No |
| rs7224610 | 17 | *HLF/HLF* | A | -0.287 | 0.137 | 0.036 | -0.258 | 0.139 | 0.064 | -0.105 | 0.129 | 0.414 | No |
| rs729761 | 6 | *VEGFA/VEGFA* | T | 0.243 | 0.128 | 0.058 | 0.254 | 0.133 | 0.055 | -0.317 | 0.122 | 0.009 | No |
| rs17786744 | 8 | *STC1/STC1* | A | 0.300 | 0.168 | 0.074 | 0.319 | 0.171 | 0.062 | -0.213 | 0.158 | 0.178 | No |
| rs1165151 | 6 | *SLC17A1/SLC17A3* | T | -0.087 | 0.055 | 0.117 | -0.055 | 0.057 | 0.327 | -0.124 | 0.052 | 0.018 | No |
| rs2941484 | 8 | *HNF4G/HNF4G* | T | -0.159 | 0.104 | 0.126 | -0.080 | 0.108 | 0.462 | -0.031 | 0.098 | 0.755 | No |
| rs11264341 | 1 | *TRIM46/PKLR* | T | -0.154 | 0.113 | 0.171 | -0.158 | 0.115 | 0.167 | 0.100 | 0.106 | 0.347 | No |
| rs1171614 | 10 | *SLC16A9/SLC16A9* | T | 0.120 | 0.093 | 0.197 | 0.077 | 0.096 | 0.422 | 0.020 | 0.089 | 0.820 | No |
| rs2231142 | 4 | *ABCG2/ABCG2* | T | 0.031 | 0.026 | 0.234 | 0.044 | 0.027 | 0.106 | -0.056 | 0.025 | 0.027 | No |
| rs2079742 | 17 | *BCAS3/C17orf82* | T | 0.163 | 0.149 | 0.275 | 0.086 | 0.153 | 0.573 | 0.214 | 0.141 | 0.130 | No |
| rs1394125 | 15 | *UBE2Q2/NRG4* | A | 0.135 | 0.137 | 0.326 | 0.135 | 0.140 | 0.334 | -0.100 | 0.130 | 0.443 | No |
| rs6598541 | 15 | *IGF1R/IGF1R* | A | -0.070 | 0.084 | 0.402 | -0.050 | 0.089 | 0.573 | -0.248 | 0.082 | 0.002 | No |
| rs478607 | 11 | *NRXN2/SLC22A12* | A | 0.110 | 0.146 | 0.449 | 0.150 | 0.150 | 0.317 | -0.188 | 0.138 | 0.173 | No |
| rs10821905 | 10 | *A1CF/ASAH2* | A | 0.085 | 0.130 | 0.514 | 0.051 | 0.132 | 0.700 | 0.019 | 0.121 | 0.876 | No |
| rs2307394 | 2 | *ORC4L/ACVR2A* | T | -0.094 | 0.151 | 0.534 | -0.071 | 0.154 | 0.643 | -0.083 | 0.143 | 0.562 | No |
| rs164009 | 17 | *QRICH2/PRPSAP1* | A | 0.097 | 0.183 | 0.597 | -0.072 | 0.186 | 0.697 | 0.097 | 0.169 | 0.568 | No |
| rs7193778 | 16 | *NFAT5/NFAT5* | T | 0.081 | 0.157 | 0.608 | -0.028 | 0.160 | 0.862 | -0.253 | 0.147 | 0.085 | No |
| rs7188445 | 16 | *MAF/MAF* | A | -0.078 | 0.175 | 0.655 | -0.019 | 0.178 | 0.916 | 0.091 | 0.163 | 0.577 | No |
| rs675209 | 6 | *RREB1/RREB1* | T | 0.035 | 0.092 | 0.704 | -0.083 | 0.094 | 0.378 | 0.056 | 0.087 | 0.525 | No |
| rs12498742 | 4 | *SLC2A9/SLC2A9* | A | 0.005 | 0.016 | 0.747 | 0.008 | 0.016 | 0.617 | -0.019 | 0.015 | 0.186 | No |
| rs10480300 | 7 | *PRKAG2/PRKAG2* | T | 0.053 | 0.178 | 0.766 | 0.072 | 0.184 | 0.697 | -0.022 | 0.169 | 0.897 | No |
| rs2078267 | 11 | *SLC22A11/SLC22A11* | T | -0.013 | 0.067 | 0.848 | -0.054 | 0.068 | 0.428 | 0.074 | 0.062 | 0.227 | No |
| rs17632159 | 5 | *TMEM171/TMEM171* | C | -0.026 | 0.155 | 0.865 | -0.113 | 0.161 | 0.481 | 0.342 | 0.145 | 0.018 | No |
| rs1471633 | 1 | *PDZK1/PDZK1* | A | -0.008 | 0.089 | 0.926 | 0.054 | 0.090 | 0.549 | -0.190 | 0.082 | 0.020 | No |
| rs742132 | 6 | *LRRC16A/LRRC16A* | A | -0.011 | 0.163 | 0.944 | -0.049 | 0.166 | 0.769 | -0.009 | 0.151 | 0.955 | No |

*GWAS summary data were obtained from the Global Lipids Genetic Consortium (GLGC).

Abbreviations: chr, chromosome; TC, total cholesterol; LDL-c, low-density lipoprotein cholesterol; HDL-c, high-density lipoprotein cholesterol.
