# Supplementary material for: Genetically determined serum urate levels and cardiovascular and other diseases in UK Biobank cohort: A phenome-wide mendelian randomization study
Source: PLoS Med. 2019 Oct 18;16(10):e1002937. doi: 10.1371/journal.pmed.1002937 (PMC6799886; doi:10.1371/journal.pmed.1002937)
Supplement: S6 Table — (DOCX) [file pmed.1002937.s009.docx]

**S6 Table. A summary of pleiotropic loci on urate and glucose.^*^**

| **SNP** | **Chr** | **Closest/GRAIL gene** | **Effect allele** |  | **Fasting glucose (n=15,234)** | | | **2hr glucose (n=58,074)** | | | **Glycoproteins (n=18,732)** | | **Pleiotropy** |
| --- | --- | --- | --- | --- | --- | --- | --- | --- | --- | --- | --- | --- | --- |
|  |  |  |  | **beta** | **se** | **p-value** | **beta** | **se** | **p-value** | **beta** | **se** | **p-value** |  |
| rs1260326 | 2 | *GCKR/GCKR* | T | 1.182 | 0.247 | 1.67E-06 | -0.416 | 0.040 | 5.57E-25 | -0.078 | 0.151 | 0.603 | Yes |
| rs6598541 | 15 | *IGF1R/IGF1R* | A | -0.682 | 0.455 | 0.134 | 0.273 | 0.075 | 2.77E-04 | 0.297 | 0.251 | 0.236 | Yes |
| rs1171614 | 10 | *SLC16A9/SLC16A9* | T | -0.297 | 0.392 | 0.448 | -0.085 | 0.057 | 0.134 | 0.747 | 0.192 | 1.01E-04 | Yes |
| rs1394125 | 15 | *UBE2Q2/NRG4* | A | -1.302 | 0.512 | 0.011 | 0.009 | 0.084 | 0.912 | -0.302 | 0.295 | 0.306 | No |
| rs17050272 | 2 | *INHBB/INHBB* | A | 1.568 | 0.622 | 0.012 | 0.135 | 0.097 | 0.165 | 0.179 | 0.298 | 0.549 | No |
| rs7224610 | 17 | *HLF/HLF* | A | 1.158 | 0.500 | 0.021 | 0.018 | 0.084 | 0.827 | 0.532 | 0.298 | 0.074 | No |
| rs478607 | 11 | *NRXN2/SLC22A12* | A | -1.083 | 0.521 | 0.038 | 0.025 | 0.088 | 0.775 | 0.189 | 0.276 | 0.493 | No |
| rs653178 | 12 | *ATXN2/PTPN11* | T | 0.833 | 0.528 | 0.114 | -0.053 | 0.089 | 0.553 | -0.044 | 0.302 | 0.883 | No |
| rs3741414 | 12 | *INHBC/INHBE* | T | -0.479 | 0.324 | 0.139 | 0.014 | 0.054 | 0.792 | 0.083 | 0.177 | 0.638 | No |
| rs6770152 | 3 | *SFMBT1/MUSTN1* | T | 0.563 | 0.396 | 0.155 | 0.044 | 0.065 | 0.498 | 0.490 | 0.226 | 0.030 | No |
| rs7188445 | 16 | *MAF/MAF* | A | -0.813 | 0.625 | 0.194 | -0.191 | 0.103 | 0.065 | -0.601 | 0.355 | 0.091 | No |
| rs10480300 | 7 | *PRKAG2/PRKAG2* | T | -0.719 | 0.656 | 0.273 | -0.313 | 0.109 | 0.004 | 0.495 | 0.409 | 0.226 | No |
| rs2231142 | 4 | *ABCG2/ABCG2* | T | -0.150 | 0.145 | 0.302 | 0.031 | 0.024 | 0.199 | 0.104 | 0.093 | 0.264 | No |
| rs1165151 | 6 | *SLC17A1/SLC17A3* | T | 0.207 | 0.207 | 0.317 | 0.068 | 0.034 | 0.042 | 0.112 | 0.116 | 0.335 | No |
| rs164009 | 17 | *QRICH2/PRPSAP1* | A | 0.655 | 0.655 | 0.317 | 0.086 | 0.110 | 0.435 | -0.040 | 0.380 | 0.915 | No |
| rs7193778 | 16 | *NFAT5/NFAT5* | T | 0.447 | 0.574 | 0.437 | 0.136 | 0.096 | 0.155 | 0.050 | 0.324 | 0.878 | No |
| rs1178977 | 7 | *BAZ1B/MLXIPL* | A | -0.340 | 0.480 | 0.479 | -0.122 | 0.080 | 0.127 | -0.268 | 0.279 | 0.338 | No |
| rs2307394 | 2 | *ORC4L/ACVR2A* | T | -0.400 | 0.571 | 0.484 | -0.191 | 0.094 | 0.042 | -0.252 | 0.339 | 0.457 | No |
| rs11264341 | 1 | *TRIM46/PKLR* | T | -0.292 | 0.417 | 0.484 | -0.075 | 0.069 | 0.275 | -0.355 | 0.222 | 0.109 | No |
| rs2078267 | 11 | *SLC22A11/SLC22A11* | T | -0.154 | 0.244 | 0.528 | -0.067 | 0.041 | 0.104 | -0.027 | 0.137 | 0.843 | No |
| rs1471633 | 1 | *PDZK1/PDZK1* | A | -0.197 | 0.311 | 0.528 | 0.036 | 0.051 | 0.478 | -0.035 | 0.173 | 0.839 | No |
| rs742132 | 6 | *LRRC16A/LRRC16A* | A | -0.371 | 0.600 | 0.536 | 0.089 | 0.097 | 0.362 | -0.219 | 0.341 | 0.521 | No |
| rs2941484 | 8 | *HNF4G/HNF4G* | T | 0.180 | 0.388 | 0.643 | 0.067 | 0.063 | 0.287 | -0.021 | 0.219 | 0.924 | No |
| rs675209 | 6 | *RREB1/RREB1* | T | -0.156 | 0.349 | 0.656 | 0.130 | 0.057 | 0.023 | 0.215 | 0.183 | 0.240 | No |
| rs2079742 | 17 | *BCAS3/C17orf82* | T | 0.235 | 0.529 | 0.657 | -0.059 | 0.090 | 0.514 | -0.622 | 0.274 | 0.023 | No |
| rs17786744 | 8 | *STC1/STC1* | A | 0.255 | 0.613 | 0.678 | 0.132 | 0.100 | 0.186 | -0.187 | 0.344 | 0.588 | No |
| rs729761 | 6 | *VEGFA/VEGFA* | T | -0.150 | 0.478 | 0.754 | -0.202 | 0.078 | 0.010 | -0.347 | 0.257 | 0.177 | No |
| rs17632159 | 5 | *TMEM171/TMEM171* | C | -0.161 | 0.579 | 0.782 | -0.050 | 0.092 | 0.587 | -0.093 | 0.317 | 0.769 | No |
| rs10821905 | 10 | *A1CF/ASAH2* | A | 0.045 | 0.453 | 0.920 | 0.079 | 0.075 | 0.294 | -0.413 | 0.241 | 0.087 | No |
| rs642803 | 11 | *OVOL1/LTBP3* | T | -0.014 | 0.442 | 0.975 | 0.014 | 0.072 | 0.847 | 0.068 | 0.249 | 0.785 | No |
| rs12498742 | 4 | *SLC2A9/SLC2A9* | A | -0.001 | 0.058 | 0.985 | 0.000 | 0.009 | 0.978 | 0.029 | 0.033 | 0.376 | No |

*GWAS summary data were obtained from the Meta-Analyses of Glucose and Insulin-related traits (MAGIC) Consortium.
