# Supplementary material for: Genetically determined serum urate levels and cardiovascular and other diseases in UK Biobank cohort: A phenome-wide mendelian randomization study
Source: PLoS Med. 2019 Oct 18;16(10):e1002937. doi: 10.1371/journal.pmed.1002937 (PMC6799886; doi:10.1371/journal.pmed.1002937)
Supplement: S7 Table — GRS, polygenic risk score. (DOCX) [file pmed.1002937.s010.docx]

**S7 Table. Association between the GRS of urate and potential confounding factors.**

| **Continuous variable** | **Mean (SD)** | **Beta (se)** | **p-value** |
| --- | --- | --- | --- |
| Age | 56.87 (7.99) | 0.010 (0.044) | 0.830 |
| BMI | 27.40 (4.76) | -0.023 (0.027) | 0.381 |
| PC1 score | -12.35 (1.61) | 0.007 (0.009) | 0.408 |
| PC2 score | 3.78 (1.50) | -0.023 (0.008) | 0.007 |
| PC3 score | -1.59 (1.58) | -0.003 (0.009) | 0.753 |
| PC4 score | 1.29 (2.94) | 0.104 (0.016) | 1.74e-10 |
| PC5 score | -0.81 (6.61) | 0.344 (0.037) | 2.20e-16 |
| **Categorical variable** | **Levels** | **F-value** | **p-value** |
| Sex | male/female | 0.476 | 0.490 |
| Assessment center | 22 centers | 3.451 | 1.41e-07 |

Abbreviations: BMI, body mass index; PC, (genetic) principal component
