# Supplementary material for: Genetically determined serum urate levels and cardiovascular and other diseases in UK Biobank cohort: A phenome-wide mendelian randomization study
Source: PLoS Med. 2019 Oct 18;16(10):e1002937. doi: 10.1371/journal.pmed.1002937 (PMC6799886; doi:10.1371/journal.pmed.1002937)
Supplement: S8 Table — GRS, polygenic risk score; TreeWAS, tree-structured phenotypic model. (DOCX) [file pmed.1002937.s011.docx]

**S8 Table. Phenotypes associated with the weighted GRS of urate in TreeWAS analysis (PP≥0.95).**

| **ICD-10 coding** | **Disease description** | **max_b^†^** | **b_ci_lhs^†^** | **b_ci_rhs^†^** | **OR (95%CI)** | **PP^*^** |
| --- | --- | --- | --- | --- | --- | --- |
| M10 | M10 Gout | 1.640 | 1.515 | 1.765 | 5.16 (4.55, 5.84) | 1.000 |
| M100 | M10.0 Idiopathic gout | 1.640 | 1.515 | 1.765 | 5.16 (4.55, 5.84) | 0.993 |
| M1007 | M10.07 Idiopathic gout (Ankle and foot) | 1.640 | 1.515 | 1.765 | 5.16 (4.55, 5.84) | 0.993 |
| M109 | M10.9 Gout, unspecified | 1.640 | 1.515 | 1.765 | 5.16 (4.55, 5.84) | 1.000 |
| M1099 | M10.99 Gout, unspecified (Site unspecified) | 1.640 | 1.515 | 1.765 | 5.16 (4.55, 5.84) | 1.000 |
| M1097 | M10.97 Gout, unspecified (Ankle and foot) | 1.640 | 1.515 | 1.765 | 5.16 (4.55, 5.84) | 1.000 |
| M1096 | M10.96 Gout, unspecified (Lower leg) | 1.640 | 1.515 | 1.765 | 5.16 (4.55, 5.84) | 1.000 |
| M1094 | M10.94 Gout, unspecified (Hand) | 1.640 | 1.515 | 1.765 | 5.16 (4.55, 5.84) | 0.993 |
| M1090 | M10.90 Gout, unspecified (Multiple sites) | 1.640 | 1.515 | 1.765 | 5.16 (4.55, 5.84) | 0.985 |
| M1001 | M10.01 Idiopathic gout (Shoulder) | 1.640 | 1.515 | 1.765 | 5.16 (4.55, 5.84) | 1.000 |
| Chapter IX | Chapter IX Diseases of the circulatory system | 0.070 | 0.055 | 0.085 | 1.07 (1.06, 1.09) | 1.000 |
| Block I10-I15 | I10-I15 Hypertensive diseases | 0.070 | 0.055 | 0.085 | 1.07 (1.06, 1.09) | 1.000 |
| I10 | I10 Essential (primary) hypertension | 0.070 | 0.055 | 0.085 | 1.07 (1.06, 1.09) | 1.000 |
| Block I20-I25 | I20-I25 Ischaemic heart diseases | 0.070 | 0.055 | 0.085 | 1.07 (1.06, 1.09) | 1.000 |
| I20 | I20 Angina pectoris | 0.070 | 0.055 | 0.085 | 1.07 (1.06, 1.09) | 0.994 |
| I209 | I20.9 Angina pectoris, unspecified | 0.070 | 0.055 | 0.085 | 1.07 (1.06, 1.09) | 0.972 |
| I21 | I21 Acute myocardial infarction | 0.070 | 0.055 | 0.085 | 1.07 (1.06, 1.09) | 0.994 |
| I219 | I21.9 Acute myocardial infarction, unspecified | 0.070 | 0.055 | 0.085 | 1.07 (1.06, 1.09) | 0.966 |
| I25 | I25 Chronic ischaemic heart disease | 0.070 | 0.055 | 0.085 | 1.07 (1.06, 1.09) | 1.000 |
| I251 | I25.1 Atherosclerotic heart disease | 0.070 | 0.055 | 0.085 | 1.07 (1.06, 1.09) | 0.999 |
| I252 | I25.2 Old myocardial infarction | 0.070 | 0.055 | 0.085 | 1.07 (1.06, 1.09) | 0.987 |
| Block I30-I52 | I30-I52 Other forms of heart disease | 0.070 | 0.055 | 0.085 | 1.07 (1.06, 1.09) | 0.999 |
| I50 | I50 Heart failure | 0.070 | 0.055 | 0.085 | 1.07 (1.06, 1.09) | 0.994 |
| I501 | I50.1 Left ventricular failure | 0.070 | 0.055 | 0.085 | 1.07 (1.06, 1.09) | 0.966 |
| Block I60-I69 | I60-I69 Cerebrovascular diseases | 0.070 | 0.055 | 0.085 | 1.07 (1.06, 1.09) | 0.991 |
| I63 | I63 Cerebral infarction | 0.070 | 0.055 | 0.085 | 1.07 (1.06, 1.09) | 0.951 |

* PP, posterior probability for the beta (β) estimate in the tree analysis not being zero.

† max_b: maximum a posteriori effect estimate (beta) and the 95% credible interval (max_b_lhs, max_b_rhs).
