# Supplementary material for: Genetically determined serum urate levels and cardiovascular and other diseases in UK Biobank cohort: A phenome-wide mendelian randomization study
Source: PLoS Med. 2019 Oct 18;16(10):e1002937. doi: 10.1371/journal.pmed.1002937 (PMC6799886; doi:10.1371/journal.pmed.1002937)
Supplement: S9 Table — MR-MoE, a mixture-of-experts machine learning framework of mendelian randomization. (DOCX) [file pmed.1002937.s012.docx]

**S9 Table. Results from MR-MoE analysis for urate and gout.**

| **Method** | **nsnp** | **beta** | **se** | **ci_low** | **ci_upp** | **pval** | **MOE^*^** |
| --- | --- | --- | --- | --- | --- | --- | --- |
| FE IVW | 31 | 1.504 | 0.081 | 1.287 | 1.722 | 3.35E-77 | 0.80 |
| Simple median | 31 | 1.708 | 0.199 | 1.318 | 2.098 | 9.18E-18 | 0.79 |
| RE IVW | 31 | 1.504 | 0.111 | 1.287 | 1.722 | 2.53E-14 | 0.77 |
| Weighted median | 31 | 1.224 | 0.118 | 0.994 | 1.455 | 2.28E-25 | 0.72 |
| Penalised median | 31 | 1.204 | 0.110 | 0.988 | 1.421 | 1.07E-27 | 0.71 |
| FE Egger | 31 | 1.400 | 0.121 | 1.070 | 1.730 | 1.02E-30 | 0.70 |
| Weighted mode | 31 | 1.221 | 0.124 | 0.979 | 1.464 | 6.06E-11 | 0.67 |
| Simple mode | 31 | 1.842 | 0.404 | 1.051 | 2.634 | 7.99E-05 | 0.65 |
| Penalised mode | 31 | 1.221 | 0.116 | 0.994 | 1.449 | 1.37E-11 | 0.56 |
| RE Egger | 31 | 1.400 | 0.168 | 1.070 | 1.730 | 3.61E-09 | 0.42 |

*A predictor for each method for how well it performs in terms of high power and low type 1 error (scaled 0-1, where 1 is best performance) for causal inference; (FE, fixed-effect; RE, random-effect; IVW, inverse variance weighted).

.
