# Supplementary material for: Genetically determined serum urate levels and cardiovascular and other diseases in UK Biobank cohort: A phenome-wide mendelian randomization study
Source: PLoS Med. 2019 Oct 18;16(10):e1002937. doi: 10.1371/journal.pmed.1002937 (PMC6799886; doi:10.1371/journal.pmed.1002937)
Supplement: S10 Table — DBP, diastolic blood pressure; MR-MoE, a mixture-of-experts machine learning framework of mendelian randomization. (DOCX) [file pmed.1002937.s013.docx]

**S10 Table. Results from MR-MoE analysis for urate and diastolic blood pressure (DBP).**

| **Method** | **nsnp** | **beta** | **se** | **ci_low** | **ci_upp** | **pval** | **MOE^*^** |
| --- | --- | --- | --- | --- | --- | --- | --- |
| Simple mode | 31 | 0.034 | 0.019 | -0.003 | 0.070 | 0.084 | 0.88 |
| Weighted mode | 31 | 0.016 | 0.007 | 0.003 | 0.029 | 0.024 | 0.80 |
| Weighted median | 31 | 0.018 | 0.007 | 0.004 | 0.032 | 0.013 | 0.79 |
| Simple median | 31 | 0.050 | 0.019 | 0.013 | 0.086 | 0.007 | 0.78 |
| Penalised median | 31 | 0.018 | 0.007 | 0.003 | 0.032 | 0.015 | 0.76 |
| Penalised mode | 31 | 0.016 | 0.007 | 0.002 | 0.029 | 0.030 | 0.71 |
| FE IVW | 31 | 0.042 | 0.006 | 0.003 | 0.081 | 1.23E-13 | 0.69 |
| FE Egger | 31 | -0.010 | 0.008 | -0.063 | 0.042 | 0.212 | 0.64 |
| RE IVW | 31 | 0.042 | 0.020 | 0.003 | 0.081 | 0.044 | 0.59 |
| RE Egger | 31 | -0.010 | 0.027 | -0.063 | 0.042 | 0.701 | 0.37 |

*A predictor for each method for how well it performs in terms of high power and low type 1 error (scaled 0-1, where 1 is best performance) for causal inference; (FE, fixed-effect; RE, random-effect; IVW, inverse variance weighted).
