# Supplementary material for: Genetically determined serum urate levels and cardiovascular and other diseases in UK Biobank cohort: A phenome-wide mendelian randomization study
Source: PLoS Med. 2019 Oct 18;16(10):e1002937. doi: 10.1371/journal.pmed.1002937 (PMC6799886; doi:10.1371/journal.pmed.1002937)
Supplement: S11 Table — MR-MoE, a mixture-of-experts machine learning framework of mendelian randomization; SBP, systemic blood pressure. (DOCX) [file pmed.1002937.s014.docx]

**S11 Table. Results from MR-MoE analysis for urate and systemic blood pressure (SBP).**

| **Method** | **nsnp** | **beta** | **se** | **ci_low** | **ci_upp** | **pval** | **MOE^*^** |
| --- | --- | --- | --- | --- | --- | --- | --- |
| Weighted mode | 31 | 0.008 | 0.007 | -0.006 | 0.023 | 0.266 | 0.72 |
| Simple mode | 31 | 0.027 | 0.030 | -0.032 | 0.086 | 0.380 | 0.66 |
| Penalised mode | 31 | 0.012 | 0.009 | -0.004 | 0.029 | 0.155 | 0.66 |
| Weighted median | 31 | 0.011 | 0.007 | -0.004 | 0.025 | 0.155 | 0.64 |
| Simple median | 31 | 0.066 | 0.017 | 0.032 | 0.100 | 1.46E-04 | 0.61 |
| Penalised median | 31 | 0.011 | 0.007 | -0.004 | 0.025 | 0.142 | 0.57 |
| FE IVW | 31 | 0.031 | 0.006 | 0.001 | 0.061 | 0.000 | 0.55 |
| RE IVW | 31 | 0.031 | 0.015 | 0.001 | 0.061 | 0.051 | 0.54 |
| FE Egger | 31 | -0.015 | 0.008 | -0.053 | 0.024 | 0.076 | 0.52 |
| RE Egger | 31 | -0.015 | 0.020 | -0.053 | 0.024 | 0.457 | 0.40 |

*A predictor for each method for how well it performs in terms of high power and low type 1 error (scaled 0-1, where 1 is best performance) for causal inference; (FE, fixed-effect; RE, random-effect; IVW, inverse variance weighted).
