# Supplementary material for: Genetically determined serum urate levels and cardiovascular and other diseases in UK Biobank cohort: A phenome-wide mendelian randomization study
Source: PLoS Med. 2019 Oct 18;16(10):e1002937. doi: 10.1371/journal.pmed.1002937 (PMC6799886; doi:10.1371/journal.pmed.1002937)
Supplement: S12 Table — CHD, coronary heart disease; MR-MoE, a mixture-of-experts machine learning framework of mendelian randomization. (DOCX) [file pmed.1002937.s015.docx]

**S12 Table. Results from MR-MoE analysis for urate and coronary heart disease (CHD).**

| **Method** | **nsnp** | **beta** | **se** | **ci_low** | **ci_upp** | **pval** | **MOE^*^** |
| --- | --- | --- | --- | --- | --- | --- | --- |
| Weighted median | 31 | 0.047 | 0.028 | -0.007 | 0.102 | 0.086 | 0.81 |
| Simple median | 31 | 0.171 | 0.055 | 0.062 | 0.280 | 0.002 | 0.78 |
| Simple mode | 31 | 0.172 | 0.073 | 0.028 | 0.315 | 0.026 | 0.77 |
| Penalised median | 31 | 0.046 | 0.029 | -0.010 | 0.102 | 0.105 | 0.76 |
| FE IVW | 31 | 0.098 | 0.022 | 0.024 | 0.172 | 7.34E-06 | 0.71 |
| Weighted mode | 31 | 0.048 | 0.025 | -0.001 | 0.097 | 0.065 | 0.71 |
| Penalised mode | 31 | 0.048 | 0.026 | -0.004 | 0.100 | 0.080 | 0.71 |
| RE IVW | 31 | 0.098 | 0.038 | 0.024 | 0.172 | 0.014 | 0.68 |
| FE Egger | 31 | -0.002 | 0.032 | -0.099 | 0.094 | 0.939 | 0.47 |
| RE Egger | 31 | -0.002 | 0.049 | -0.099 | 0.094 | 0.961 | 0.35 |

*A predictor for each method for how well it performs in terms of high power and low type 1 error (scaled 0-1, where 1 is best performance) for causal inference; (FE, fixed-effect; RE, random-effect; IVW, inverse variance weighted).
