# Supplementary material for: Genetically determined serum urate levels and cardiovascular and other diseases in UK Biobank cohort: A phenome-wide mendelian randomization study
Source: PLoS Med. 2019 Oct 18;16(10):e1002937. doi: 10.1371/journal.pmed.1002937 (PMC6799886; doi:10.1371/journal.pmed.1002937)
Supplement: S13 Table — MR-MoE, a mixture-of-experts machine learning framework of mendelian randomization. (DOCX) [file pmed.1002937.s016.docx]

**S13 Table. Results from MR-MoE analysis for urate and myocardial infarction (MI).**

| **Method** | **nsnp** | **beta** | **se** | **ci_low** | **ci_upp** | **pval** | **MOE^*^** |
| --- | --- | --- | --- | --- | --- | --- | --- |
| Weighted median | 31 | 0.058 | 0.030 | -0.001 | 0.117 | 0.055 | 0.81 |
| Simple mode | 31 | 0.215 | 0.083 | 0.053 | 0.377 | 0.014 | 0.79 |
| FE IVW | 31 | 0.105 | 0.024 | 0.024 | 0.186 | 1.45E-05 | 0.78 |
| Simple median | 31 | 0.192 | 0.057 | 0.080 | 0.304 | 0.001 | 0.76 |
| Penalised mode | 31 | 0.047 | 0.030 | -0.012 | 0.106 | 0.125 | 0.73 |
| Penalised median | 31 | 0.056 | 0.030 | -0.003 | 0.115 | 0.064 | 0.72 |
| Weighted mode | 31 | 0.047 | 0.029 | -0.009 | 0.104 | 0.112 | 0.72 |
| RE IVW | 31 | 0.105 | 0.041 | 0.024 | 0.186 | 0.017 | 0.65 |
| FE Egger | 31 | -0.001 | 0.035 | -0.108 | 0.106 | 0.978 | 0.46 |
| RE Egger | 31 | -0.001 | 0.055 | -0.108 | 0.106 | 0.986 | 0.40 |

*A predictor for each method for how well it performs in terms of high power and low type 1 error (scaled 0-1, where 1 is best performance) for causal inference; (FE, fixed-effect; RE, random-effect; IVW, inverse variance weighted).
