# Supplementary material for: Genetically determined serum urate levels and cardiovascular and other diseases in UK Biobank cohort: A phenome-wide mendelian randomization study
Source: PLoS Med. 2019 Oct 18;16(10):e1002937. doi: 10.1371/journal.pmed.1002937 (PMC6799886; doi:10.1371/journal.pmed.1002937)
Supplement: S14 Table — MR-MoE, a mixture-of-experts machine learning framework of mendelian randomization; TC, total cholesterol. (DOCX) [file pmed.1002937.s017.docx]

**S14 Table. Results from MR-MoE analysis for urate and total cholesterol (TC).**

| **Method** | **nsnp** | **beta** | **se** | **ci_low** | **ci_upp** | **pval** | **MOE^*^** |
| --- | --- | --- | --- | --- | --- | --- | --- |
| Simple median | 31 | 0.005 | 0.026 | -0.046 | 0.056 | 0.848 | 0.87 |
| RE IVW | 31 | 0.028 | 0.036 | -0.042 | 0.098 | 0.440 | 0.85 |
| RE Egger | 31 | 0.050 | 0.053 | -0.053 | 0.154 | 0.348 | 0.82 |
| Weighted median | 31 | 0.011 | 0.014 | -0.017 | 0.038 | 0.452 | 0.81 |
| Penalised mode | 31 | 0.011 | 0.013 | -0.016 | 0.037 | 0.433 | 0.80 |
| Simple mode | 31 | 0.045 | 0.043 | -0.039 | 0.129 | 0.302 | 0.79 |
| Penalised median | 31 | 0.011 | 0.014 | -0.017 | 0.039 | 0.442 | 0.77 |
| Weighted mode | 31 | 0.011 | 0.012 | -0.014 | 0.035 | 0.398 | 0.76 |
| FE Egger | 31 | 0.050 | 0.016 | -0.053 | 0.154 | 0.001 | 0.61 |
| FE IVW | 31 | 0.028 | 0.011 | -0.042 | 0.098 | 0.009 | 0.52 |

*A predictor for each method for how well it performs in terms of high power and low type 1 error (scaled 0-1, where 1 is best performance) for causal inference; (FE, fixed-effect; RE, random-effect; IVW, inverse variance weighted).
