# Supplementary material for: Genetically determined serum urate levels and cardiovascular and other diseases in UK Biobank cohort: A phenome-wide mendelian randomization study
Source: PLoS Med. 2019 Oct 18;16(10):e1002937. doi: 10.1371/journal.pmed.1002937 (PMC6799886; doi:10.1371/journal.pmed.1002937)
Supplement: S15 Table — HDL-c, high-density lipoprotein cholesterol; MR-MoE, a mixture-of-experts machine learning framework of mendelian randomization. (DOCX) [file pmed.1002937.s018.docx]

**S15 Table. Results from MR-MoE analysis for urate and high-density lipoprotein cholesterol (HDL_c).**

| **Method** | **nsnp** | **beta** | **se** | **ci_low** | **ci_upp** | **pval** | **MOE^*^** |
| --- | --- | --- | --- | --- | --- | --- | --- |
| Penalised mode | 31 | -0.030 | 0.015 | -0.059 | 0.001 | 0.058 | 0.74 |
| Weighted mode | 31 | -0.030 | 0.014 | -0.056 | -0.003 | 0.038 | 0.72 |
| RE IVW | 31 | -0.075 | 0.026 | -0.125 | -0.024 | 0.007 | 0.70 |
| RE Egger | 31 | -0.035 | 0.037 | -0.108 | 0.039 | 0.361 | 0.69 |
| Penalised median | 31 | -0.020 | 0.014 | -0.048 | 0.007 | 0.150 | 0.69 |
| Simple mode | 31 | -0.021 | 0.048 | -0.115 | 0.073 | 0.669 | 0.69 |
| Weighted median | 31 | -0.021 | 0.015 | -0.050 | 0.009 | 0.166 | 0.60 |
| Simple median | 31 | -0.083 | 0.032 | -0.145 | -0.021 | 0.009 | 0.48 |
| FE Egger | 31 | -0.035 | 0.015 | -0.108 | 0.039 | 0.021 | 0.43 |
| FE IVW | 31 | -0.075 | 0.010 | -0.125 | -0.024 | 4.09E-13 | 0.42 |

*A predictor for each method for how well it performs in terms of high power and low type 1 error (scaled 0-1, where 1 is best performance) for causal inference; (FE, fixed-effect; RE, random-effect; IVW, inverse variance weighted).
