# Supplementary material for: Genetically determined serum urate levels and cardiovascular and other diseases in UK Biobank cohort: A phenome-wide mendelian randomization study
Source: PLoS Med. 2019 Oct 18;16(10):e1002937. doi: 10.1371/journal.pmed.1002937 (PMC6799886; doi:10.1371/journal.pmed.1002937)
Supplement: S16 Table — LDL-c, low-density lipoprotein cholesterol; MR-MoE, a mixture-of-experts machine learning framework of mendelian randomization. (DOCX) [file pmed.1002937.s019.docx]

**S16 Table. Results from MR-MoE analysis for urate and low-density lipoprotein cholesterol (LDL-c).**

| **Method** | **nsnp** | **beta** | **se** | **ci_low** | **ci_upp** | **pval** | **MOE^*^** |
| --- | --- | --- | --- | --- | --- | --- | --- |
| Weighted median | 31 | 0.014 | 0.015 | -0.014 | 0.043 | 0.335 | 0.85 |
| RE IVW | 31 | 0.011 | 0.023 | -0.034 | 0.057 | 0.627 | 0.84 |
| Simple median | 31 | -0.049 | 0.029 | -0.105 | 0.007 | 0.089 | 0.78 |
| Weighted mode | 31 | 0.011 | 0.014 | -0.016 | 0.037 | 0.428 | 0.78 |
| Penalised mode | 31 | 0.011 | 0.015 | -0.018 | 0.039 | 0.460 | 0.73 |
| Penalised median | 31 | 0.012 | 0.015 | -0.018 | 0.041 | 0.438 | 0.72 |
| Simple mode | 31 | -0.038 | 0.043 | -0.122 | 0.046 | 0.377 | 0.72 |
| RE Egger | 31 | 0.041 | 0.034 | -0.025 | 0.107 | 0.230 | 0.71 |
| FE IVW | 31 | 0.011 | 0.011 | -0.034 | 0.057 | 0.298 | 0.70 |
| FE Egger | 31 | 0.041 | 0.016 | -0.025 | 0.107 | 0.010 | 0.53 |

*A predictor for each method for how well it performs in terms of high power and low type 1 error (scaled 0-1, where 1 is best performance) for causal inference; (FE, fixed-effect; RE, random-effect; IVW, inverse variance weighted).
