# Supplementary material for: Genetically determined serum urate levels and cardiovascular and other diseases in UK Biobank cohort: A phenome-wide mendelian randomization study
Source: PLoS Med. 2019 Oct 18;16(10):e1002937. doi: 10.1371/journal.pmed.1002937 (PMC6799886; doi:10.1371/journal.pmed.1002937)
Supplement: S17 Table — MR-MoE, a mixture-of-experts machine learning framework of mendelian randomization. (DOCX) [file pmed.1002937.s020.docx]

**S17 Table. Results from MR-MoE analysis for urate and ischaemic stroke (IS).**

| **Method** | **nsnp** | **beta** | **se** | **ci_low** | **ci_upp** | **pval** | **MOE^*^** |
| --- | --- | --- | --- | --- | --- | --- | --- |
| RE IVW | 31 | 0.029 | 0.052 | -0.073 | 0.131 | 0.586 | 0.81 |
| Simple median | 31 | 0.021 | 0.083 | -0.141 | 0.183 | 0.801 | 0.81 |
| Simple mean | 31 | 0.021 | 0.045 | -0.068 | 0.110 | 0.644 | 0.80 |
| Weighted mode | 31 | -0.018 | 0.047 | -0.110 | 0.074 | 0.699 | 0.74 |
| Weighted median | 31 | -0.011 | 0.049 | -0.107 | 0.086 | 0.829 | 0.72 |
| Penalised median | 31 | -0.012 | 0.046 | -0.103 | 0.079 | 0.796 | 0.71 |
| FE IVW | 31 | 0.029 | 0.038 | -0.073 | 0.131 | 0.447 | 0.65 |
| Penalised mode | 31 | -0.018 | 0.048 | -0.113 | 0.076 | 0.706 | 0.65 |
| Simple mode | 31 | -0.004 | 0.131 | -0.260 | 0.252 | 0.975 | 0.61 |
| FE Egger | 31 | -0.024 | 0.055 | -0.174 | 0.126 | 0.665 | 0.60 |
| RE Egger | 31 | -0.024 | 0.076 | -0.174 | 0.126 | 0.757 | 0.60 |

*A predictor for each method for how well it performs in terms of high power and low type 1 error (scaled 0-1, where 1 is best performance) for causal inference; (FE, fixed-effect; RE, random-effect; IVW, inverse variance weighted).
