# Supplementary material for: Genetically determined serum urate levels and cardiovascular and other diseases in UK Biobank cohort: A phenome-wide mendelian randomization study
Source: PLoS Med. 2019 Oct 18;16(10):e1002937. doi: 10.1371/journal.pmed.1002937 (PMC6799886; doi:10.1371/journal.pmed.1002937)
Supplement: S18 Table — GRS, polygenic risk score. (DOCX) [file pmed.1002937.s021.docx]

**S18 Table. Sensitivity analysis by using the GRS of genetic polymorphisms involving in renal handling of urate.**

| **Phecode** | **Disease outcomes** | **n_cases** | **n_controls** | **beta** | **se** | **OR (95%CI)** | **p-value** |
| --- | --- | --- | --- | --- | --- | --- | --- |
| *GRS of 7 SNPs involved in renal handling of urate* | | | | | | | |
| 274.1 | Gout | 2,532 | 335,108 | 1.686 | 0.083 | 5.40 (4.59, 6.36) | 3.04E-91 |
| 714 | Inflammatory polyarthropathies | 15,408 | 320,862 | 0.245 | 0.031 | 1.28 (1.20, 1.36) | 5.55E-15 |
| 716.9 | Arthropathy | 59,849 | 277,595 | 0.071 | 0.017 | 1.07 (1.04, 1.11) | 3.17E-05 |
| 709.7 | Unspecified diffuse connective tissue disease | 89,962 | 245,976 | 0.061 | 0.015 | 1.06 (1.03, 1.09) | 3.53E-05 |
| 274.11 | Gouty arthropathy | 88 | 335,108 | 1.800 | 0.445 | 6.05 (2.53, 14.47) | 5.24E-05 |
| *GRS of the remaining 24 SNPs* | | | | | | | |
| 274.1 | Gout | 2,532 | 335,108 | 1.752 | 0.140 | 5.77 (4.38, 7.59) | 7.94E-36 |
| 401 | Hypertension | 63,694 | 274,477 | 0.272 | 0.033 | 1.31 (1.23, 1.40) | 9.10E-17 |
| 401.1 | Essential hypertension | 63,442 | 274,477 | 0.271 | 0.033 | 1.31 (1.23, 1.40) | 1.44E-16 |
| 411.4 | Coronary atherosclerosis | 25,795 | 311,554 | 0.267 | 0.047 | 1.31 (1.19, 1.43) | 1.50E-08 |
| 411.8 | Chronic ischemic heart disease | 25,567 | 311,554 | 0.259 | 0.047 | 1.30 (1.18, 1.42) | 4.32E-08 |
| 411 | Ischemic Heart Disease | 25,617 | 311,554 | 0.258 | 0.047 | 1.29 (1.18, 1.42) | 5.04E-08 |
| 411.2 | Myocardial infarction | 9,829 | 311,554 | 0.394 | 0.074 | 1.48 (1.28, 1.71) | 8.85E-08 |
| 459.9 | Circulatory disease | 107,298 | 230,622 | 0.137 | 0.027 | 1.15 (1.09, 1.21) | 4.46E-07 |
| 272.11 | Hypercholesterolemia | 27,040 | 308,948 | 0.223 | 0.046 | 1.25 (1.14, 1.37) | 9.65E-07 |
| 714 | Inflammatory polyarthropathies | 15,408 | 320,862 | 0.259 | 0.058 | 1.30 (1.16, 1.45) | 8.03E-06 |
| 244.4 | Hypothyroidism | 11,446 | 324,000 | 0.291 | 0.067 | 1.34 (1.17, 1.52) | 1.38E-05 |
