# Supplementary material for: Genetically determined serum urate levels and cardiovascular and other diseases in UK Biobank cohort: A phenome-wide mendelian randomization study
Source: PLoS Med. 2019 Oct 18;16(10):e1002937. doi: 10.1371/journal.pmed.1002937 (PMC6799886; doi:10.1371/journal.pmed.1002937)
Supplement: S19 Table — (DOCX) [file pmed.1002937.s022.docx]

**S19 Table. Sensitivity analysis by including pleiotropic loci on metabolic traits.**

| **Disease outcomes** | **GRS of all-urate loci (n=31)** | | | **GRS of urate-specific loci (n=14)** | | | **GRS of urate-obesity pleiotropic loci (n=10)** | | | **GRS of urate-BP pleiotropic loci (n=10)** | | | **GRS of urate-lipid pleiotropic loci (GRS=6)** | | | **GRS of urate-glucose pleiotropic loci (GRS=3)** | | |
| --- | --- | --- | --- | --- | --- | --- | --- | --- | --- | --- | --- | --- | --- | --- | --- | --- | --- | --- |
|  | **OR (95%CI)** | **p-value** | **FDR** | **OR (95%CI)** | **p-value** | **FDR** | **OR (95%CI)** | **p-value** | **FDR** | **OR (95%CI)** | **p-value** | **FDR** | **OR (95%CI)** | **p-value** | **FDR** | **OR (95%CI)** | **p-value** | **FDR** |
| Gout | 5.37 (4.67, 6.18) | 4.27E-123 | TRUE | 3.77 (3.19, 4.46) | 4.42E-54 | TRUE | 12.82 (9.91, 16.59) | 5.10E-84 | TRUE | 5.15 (3.26, 8.15) | 2.32E-12 | TRUE | 9.52 (6.05, 14.99) | 2.19E-22 | TRUE | 10.83 (6.37, 18.41) | 1.38E-18 | TRUE |
| Inflammatory polyarthropathies | 1.27 (1.21, 1.34) | 4.97E-19 | TRUE | 1.22 (1.15, 1.30) | 6.45E-10 | TRUE | 1.57 (1.40, 1.76) | 3.39E-14 | TRUE | 1.52 (1.26, 1.84) | 1.27E-05 | TRUE | 1.52 (1.26, 1.83) | 1.25E-05 | TRUE | 1.33 (1.07, 1.66) | 0.010 | FALSE |
| Hypertension | 1.07 (1.05, 1.11) | 6.02E-07 | TRUE | 1.03 (1.00, 1.07) | 0.075 | FALSE | 1.14 (1.06, 1.22) | 1.42E-04 | TRUE | 1.72 (1.55, 1.92) | 2.13E-23 | TRUE | 1.45 (1.31, 1.61) | 3.99E-12 | TRUE | 1.10 (0.97, 1.24) | 0.138 | FALSE |
| Essential hypertension | 1.08 (1.05, 1.11) | 6.26E-07 | TRUE | 1.03 (1.00, 1.07) | 0.074 | FALSE | 1.14 (1.07, 1.22) | 1.37E-04 | TRUE | 1.72 (1.55, 1.91) | 2.87E-23 | TRUE | 1.45 (1.31, 1.61) | 4.08E-12 | TRUE | 1.10 (0.97, 1.24) | 0.146 | FALSE |
| Coronary atherosclerosis | 1.10 (1.05, 1.14) | 1.17E-05 | TRUE | 1.05 (1.00, 1.11) | 0.052 | FALSE | 1.18 (1.07, 1.30) | 5.96E-04 | FALSE | 1.38 (1.18, 1.61) | 3.37E-05 | TRUE | 1.80 (1.55, 2.09) | 1.35E-14 | TRUE | 1.45 (1.21, 1.72) | 3.89E-05 | TRUE |
| Gouty arthropathy | 5.10 (2.45, 10.66) | 1.39E-05 | TRUE | 4.38 (1.77, 10.82) | 0.001 | FALSE | 9.83 (2.50, 38.67) | 1.08E-03 | FALSE | 1.94 (0.17, 21.86) | 0.592 | FALSE | 3.28 (0.30, 36.00) | 0.331 | FALSE | 12.57 (0.75, 209.57) | 0.078 | FALSE |
| Chronic Ischaemic heart disease | 1.09 (1.05, 1.14) | 1.52E-05 | TRUE | 1.05 (1.00, 1.10) | 0.057 | FALSE | 1.18 (1.07, 1.30) | 5.79E-04 | FALSE | 1.37 (1.18, 1.59) | 5.49E-05 | TRUE | 1.79 (1.54, 2.09) | 2.64E-14 | TRUE | 1.44 (1.20, 1.71) | 5.81E-05 | TRUE |
| Ischaemic Heart Disease | 1.09 (1.05, 1.14) | 1.73E-05 | TRUE | 1.05 (1.00, 1.10) | 0.060 | FALSE | 1.18 (1.07, 1.30) | 6.64E-04 | FALSE | 1.37 (1.17, 1.59) | 5.61E-05 | TRUE | 1.79 (1.54, 2.08) | 3.61E-14 | TRUE | 1.43 (1.20, 1.71) | 6.58E-05 | TRUE |
| Myocardial infarction | 1.14 (1.07, 1.22) | 5.23E-05 | TRUE | 1.05 (0.97, 1.14) | 0.205 | FALSE | 1.30 (1.12, 1.50) | 4.54E-04 | FALSE | 1.65 (1.30, 2.09) | 3.41E-05 | TRUE | 2.31 (1.83, 2.91) | 2.27E-12 | TRUE | 1.75 (1.33, 2.30) | 6.10E-05 | TRUE |
| Pyogenic arthritis | 2.10 (1.41, 3.13) | 2.87E-04 | TRUE | 1.73 (1.07, 2.78) | 0.024 | FALSE | 2.66 (1.17, 6.08) | 2.00E-02 | FALSE | 1.71 (0.43, 6.80) | 0.444 | FALSE | 9.58 (2.44, 37.68) | 0.001 | FALSE | 6.13 (1.24, 30.22) | 0.026 | FALSE |
| Circulatory disease | 1.04 (1.02, 1.07) | 3.29E-04 | TRUE | 1.02 (0.99, 1.05) | 0.258 | FALSE | 1.10 (1.04, 1.16) | 6.94E-04 | FALSE | 1.31 (1.20, 1.43) | 1.59E-09 | TRUE | 1.29 (1.18, 1.41) | 9.47E-09 | TRUE | 1.17 (1.05, 1.29) | 0.003 | FALSE |
| Disorders of metabolism | 1.07 (1.03, 1.11) | 3.33E-04 | TRUE | 1.03 (0.99, 1.08) | 0.157 | FALSE | 1.17 (1.08, 1.27) | 1.06E-04 | TRUE | 1.12 (0.98, 1.27) | 0.100 | FALSE | 1.58 (1.39, 1.80) | 3.09E-12 | TRUE | 1.52 (1.30, 1.76) | 6.35E-08 | TRUE |
| Hypercholesterolemia | 1.08 (1.04, 1.12) | 3.34E-04 | TRUE | 1.00 (0.95, 1.05) | 0.913 | FALSE | 1.32 (1.20, 1.45) | 3.53E-09 | TRUE | 1.23 (1.06, 1.43) | 0.006 | FALSE | 1.90 (1.64, 2.20) | 8.73E-18 | TRUE | 1.84 (1.55, 2.19) | 3.01E-12 | TRUE |
