# Supplementary material for: Genetically determined serum urate levels and cardiovascular and other diseases in UK Biobank cohort: A phenome-wide mendelian randomization study
Source: PLoS Med. 2019 Oct 18;16(10):e1002937. doi: 10.1371/journal.pmed.1002937 (PMC6799886; doi:10.1371/journal.pmed.1002937)
Supplement: S20 Table — (DOCX) [file pmed.1002937.s023.docx]

**S20 Table. Sensitivity analysis by excluding the pleiotropic loci of metabolic traits.**

| **Disease outcomes** | **GRS of all urate loci (n=31)** | | | **GRS of loci without pleiotropy on obesity (n=21)** | | | **GRS of loci without pleiotropy on BP (n=21)** | | | **GRS of loci without pleiotropy on lipids (n=25)** | | | **GRS of loci without pleiotropy on glucose (n=28)** | | |
| --- | --- | --- | --- | --- | --- | --- | --- | --- | --- | --- | --- | --- | --- | --- | --- |
|  | **OR (95%CI)** | **p-value** | **FDR** | **OR (95%CI)** | **p-value** | **FDR** | **OR (95%CI)** | **p-value** | **FDR** | **OR (95%CI)** | **p-value** | **FDR** | **OR (95%CI)** | **p-value** | **FDR** |
| Gout | 5.37 (4.67, 6.18) | 4.27E-123 | TRUE | 3.89 (3.32, 4.56) | 1.01E-62 | TRUE | 5.42 (4.68, 6.28) | 6.00E-113 | TRUE | 5.07 (4.38, 5.86) | 5.27E-105 | TRUE | 5.09 (4.41, 5.88) | 1.31E-108 | TRUE |
| Inflammatory polyarthropathies | 1.27 (1.21, 1.34) | 4.97E-19 | TRUE | 1.21 (1.14, 1.28) | 6.43E-10 | TRUE | 1.26 (1.19, 1.33) | 1.02E-15 | TRUE | 1.26 (1.19, 1.33) | 1.16E-15 | TRUE | 1.27 (1.20, 1.34) | 1.34E-17 | TRUE |
| Hypertension | 1.07 (1.05, 1.11) | 6.02E-07 | TRUE | 1.07 (1.03, 1.10) | 2.31E-04 | FALSE | 1.04 (1.01, 1.07) | 2.24E-02 | FALSE | 1.05 (1.02, 1.09) | 0.002 | FALSE | 1.08 (1.05, 1.11) | 1.82E-06 | TRUE |
| Essential hypertension | 1.08 (1.05, 1.11) | 6.26E-07 | TRUE | 1.07 (1.03, 1.10) | 2.44E-04 | FALSE | 1.04 (1.01, 1.07) | 2.23E-02 | FALSE | 1.05 (1.02, 1.09) | 0.002 | FALSE | 1.08 (1.05, 1.11) | 1.83E-06 | TRUE |
| Coronary atherosclerosis | 1.10 (1.05, 1.14) | 1.17E-05 | TRUE | 1.08 (1.03, 1.13) | 0.001 | FALSE | 1.08 (1.03, 1.13) | 7.82E-04 | FALSE | 1.05 (1.01, 1.10) | 0.022 | FALSE | 1.08 (1.03, 1.13) | 4.88E-04 | FALSE |
| Gouty arthropathy | 5.10 (2.45, 10.66) | 1.39E-05 | TRUE | 4.04 (1.73, 9.44) | 0.001 | FALSE | 5.69 (2.61, 12.37) | 1.19E-05 | TRUE | 5.36 (2.47, 11.63) | 2.16E-05 | TRUE | 4.78 (2.24, 10.21) | 5.37E-05 | TRUE |
| Chronic Ischaemic heart disease | 1.09 (1.05, 1.14) | 1.52E-05 | TRUE | 1.08 (1.03, 1.13) | 0.002 | FALSE | 1.08 (1.03, 1.13) | 8.61E-04 | FALSE | 1.05 (1.01, 1.10) | 0.024 | FALSE | 1.08 (1.03, 1.13) | 5.58E-04 | FALSE |
| Ischaemic Heart Disease | 1.09 (1.05, 1.14) | 1.73E-05 | TRUE | 1.08 (1.03, 1.13) | 0.002 | FALSE | 1.08 (1.03, 1.13) | 9.51E-04 | FALSE | 1.05 (1.01, 1.10) | 0.026 | FALSE | 1.08 (1.03, 1.13) | 6.04E-04 | FALSE |
| Myocardial infarction | 1.14 (1.07, 1.22) | 5.23E-05 | TRUE | 1.11 (1.03, 1.20) | 0.006 | FALSE | 1.11 (1.04, 1.19) | 0.003 | FALSE | 1.08 (1.01, 1.16) | 0.033 | FALSE | 1.12 (1.04, 1.20) | 0.002 | FALSE |
| Pyogenic arthritis | 2.10 (1.41, 3.13) | 2.87E-04 | TRUE | 1.96 (1.24, 3.09) | 0.004 | FALSE | 2.14 (1.41, 3.26) | 3.73E-04 | FALSE | 1.82 (1.20, 2.77) | 0.005 | FALSE | 1.95 (1.29, 2.95) | 0.001 | FALSE |
| Circulatory disease | 1.04 (1.02, 1.07) | 3.29E-04 | TRUE | 1.03 (1.01, 1.06) | 0.020 | FALSE | 1.03 (1.00, 1.05) | 0.048 | FALSE | 1.03 (1.00, 1.05) | 0.041 | FALSE | 1.04 (1.01, 1.07) | 0.003 | FALSE |
| Disorders of metabolism | 1.07 (1.03, 1.11) | 3.33E-04 | TRUE | 1.04 (1.00, 1.09) | 0.038 | FALSE | 1.07 (1.03, 1.11) | 0.001 | FALSE | 1.03 (0.99, 1.07) | 0.094 | FALSE | 1.05 (1.01, 1.09) | 0.019 | FALSE |
| Hypercholesterolemia | 1.08 (1.04, 1.12) | 3.34E-04 | TRUE | 1.03 (0.98, 1.07) | 0.293 | FALSE | 1.07 (1.02, 1.12) | 0.003 | FALSE | 1.03 (0.98, 1.07) | 0.233 | FALSE | 1.04 (1.00, 1.09) | 0.051 | FALSE |
