# Supplementary figures and images for: Genetically determined serum urate levels and cardiovascular and other diseases in UK Biobank cohort: A phenome-wide mendelian randomization study
Source: PLoS Med. 2019 Oct 18;16(10):e1002937. doi: 10.1371/journal.pmed.1002937 (PMC6799886; doi:10.1371/journal.pmed.1002937)

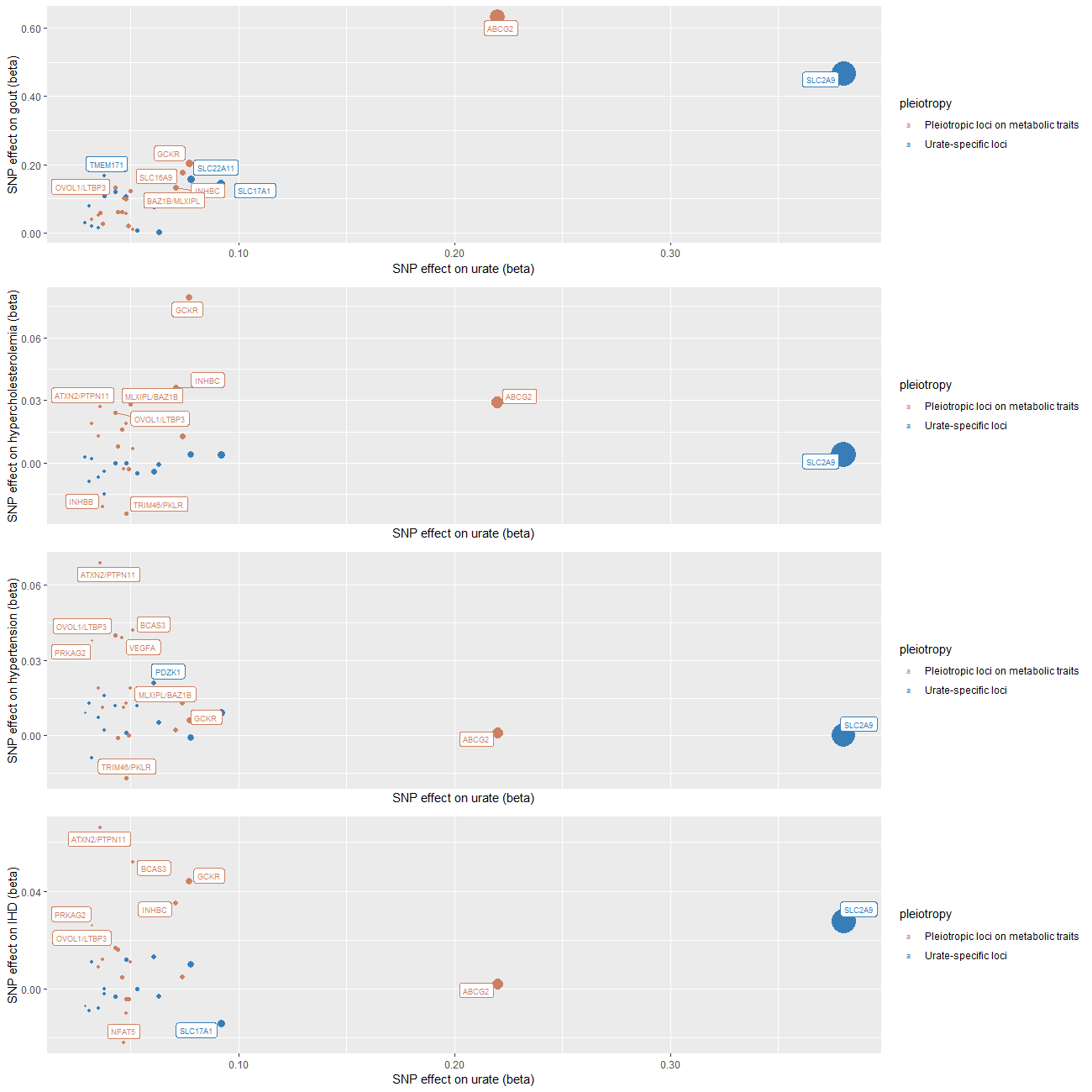

Supplement: S1 Fig — The size of the points was scaled to be inversely proportional to the standard errors of the effect sizes. Two urate transporter genes (SLC2A9 and ABCG2) are recognized as the leading loci driving the association with gout, the GCKR gene is the leading locus driving the association with hypercholesterolemia, and the PTPN11/ATXN2 gene is the leading locus driving the association with hypertension and ischemic heart diseases. (TIF) [file pmed.1002937.s024.tif]
